# CCCView: A Knowledge Platform for Integrated Analysis of Spatial Transcriptomic Cell–Cell Communication

**DOI:** 10.3390/ijms27209382

**Published:** 2026-10-21

**Authors:** Ziyi Qi, Chenyang Wu, Qiheng Qian, Ziyang Zeng, Jingfa Xiao, Dongyu Zhao

**Affiliations:** 1 Department of Biomedical Informatics, School of Basic Medical Sciences, Peking University, Beijing 100191, China; qiziyi@stu.pku.edu.cn (Z.Q.); 1810305335@pku.edu.cn (C.W.); zengziyang2005@163.com (Z.Z.); 2 State Key Laboratory of Vascular Homeostasis and Remodeling, Peking University, Beijing 100191, China; 3 National Genomics Data Center, China National Center for Bioinformation, Beijing 100101, China; qianqiheng2018m@big.ac.cn; 4 Beijing Institute of Genomics, Chinese Academy of Sciences, Beijing 100101, China; 5 University of Chinese Academy of Sciences, Beijing 100049, China

**Keywords:** spatial transcriptomics, cell–cell communication, ligand–receptor interaction, spatial omics, knowledge platform, multi-sample analysis, Tweedie regression

## Abstract

Spatial transcriptomics enables investigation of cell–cell communication (CCC) while preserving spatial context, yet large-scale resources that organize spatial CCC results across studies and make them readily searchable, visualizable, and comparable remain limited. Here, we present CCCView, a knowledge platform integrating 699 spatial transcriptomics samples and 7,473,444 inferred CCC interactions across 24 tissues, 182 annotated cell types, and 27 biological conditions in human and mouse. CCCView uses a unified cell-type annotation workflow and organizes results from three complementary CCC-inference approaches—COMMOT, CellNEST, and SpatialDM—while preserving method-specific outputs. The platform provides interactive search, browsing, visualization, disease-oriented exploration, data download, and online analysis functions. For cross-sample exploration, CCCView also includes MuCIA, a Tweedie regression-based statistical module for comparing inferred interaction strength across user-defined sample groups. A glioblastoma use case illustrates how CCCView can connect spatial cell-type context, inferred communication patterns, and condition-associated signals to generate biologically interpretable hypotheses. CCCView provides an integrated resource for systematic exploration of spatial transcriptomic CCC and for reuse of precomputed communication results across heterogeneous datasets.

## 1. Introduction

Cell–cell communication (CCC) coordinates tissue homeostasis, development, immune responses, and disease progression through direct cell–cell contact and secreted signaling molecules such as cytokines and hormones [1,2,3,4,5]. Dysregulated communication networks have been implicated in cancer, neurodegenerative disorders, and immune dysfunction [6]. For example, CCL5-CCR5 signaling can contribute to the recruitment and activity of immunosuppressive cells in the tumor microenvironment [7,8]. Systematic characterization of CCC is therefore important for understanding how cellular states are coordinated within complex tissues.

Transcriptome-based CCC inference commonly identifies candidate signaling relationships from the expression of ligands, receptors, and related pathway components. Single-cell RNA sequencing (scRNA-seq) has substantially improved the cellular resolution of these analyses and has enabled methods such as CellChat and NicheNet [3,9,10,11,12]. However, dissociation-based scRNA-seq does not retain the native spatial organization of cells, limiting the ability to distinguish interactions that are transcriptionally plausible from those that are also spatially plausible. Data sparsity and method-specific assumptions can further affect inferred communication networks [13,14].

Spatial transcriptomics (ST) addresses part of this limitation by measuring gene expression while preserving spatial coordinates [15,16,17,18,19,20,21]. The rapid expansion of ST has led to large public resources, including STOmicsDB [22], SODB [23], and Aquila [24], as well as specialized computational methods for spatial CCC inference, including COMMOT [25], CellNEST [26], SpatialDM [27], DeepTalk [28], SpaOTsc [29], and SpaTalk [30]. Existing spatial-omics databases provide broad access to datasets and analysis functions, but large-scale resources dedicated to harmonized CCC inference, cross-sample exploration, and comparative analysis remain limited.

To address this gap, we developed CCCView, a knowledge platform for integrated organization, exploration, and analysis of spatial transcriptomic CCC. CCCView currently contains 7,473,444 inferred interactions from 699 human and mouse samples spanning 24 tissues, 182 annotated cell types, and 27 biological conditions. A unified annotation workflow based on Cell BLAST (v0.5.1) [31] and Tangram [32] is combined with three complementary CCC-inference methods—COMMOT, CellNEST, and SpatialDM. Rather than collapsing heterogeneous method outputs into a single score, CCCView preserves their method-specific results within a common data organization and interface. The platform provides interactive search, browsing, visualization, disease-oriented exploration, data download, and online analysis functions. It also includes MuCIA as a statistical module for cross-sample comparison of inferred interaction strength. Together, these components are intended to make large-scale spatial CCC results easier to access, compare, and reuse for biological hypothesis generation.

## 2. Results

CCCView is freely accessible at http://cccview.bjmu.edu.cn/. CCCView provides seven main interfaces: Home, Search, Browse, Tools, Disease, Submission and Document. These interfaces allow users to explore cell–cell communication (CCC) data in various ways. The system contains detailed information from 699 high-quality spatial transcriptome samples, encompassing 7.4 million CCC interactions across human and mouse species. The current database release is designated CCCView v1.0, with a data collection cutoff of September 2026. Comprehensive metadata for the 699 samples, including species, tissue, experimental conditions, spatial transcriptomics technologies, dataset information, literature references, and source URLs, are provided in Supplementary Table S3 to improve data provenance and traceability. Where available, the source URLs link to original study accessions or publications; otherwise, links to relevant public data repositories or providers are supplied.

**Figure 1:**
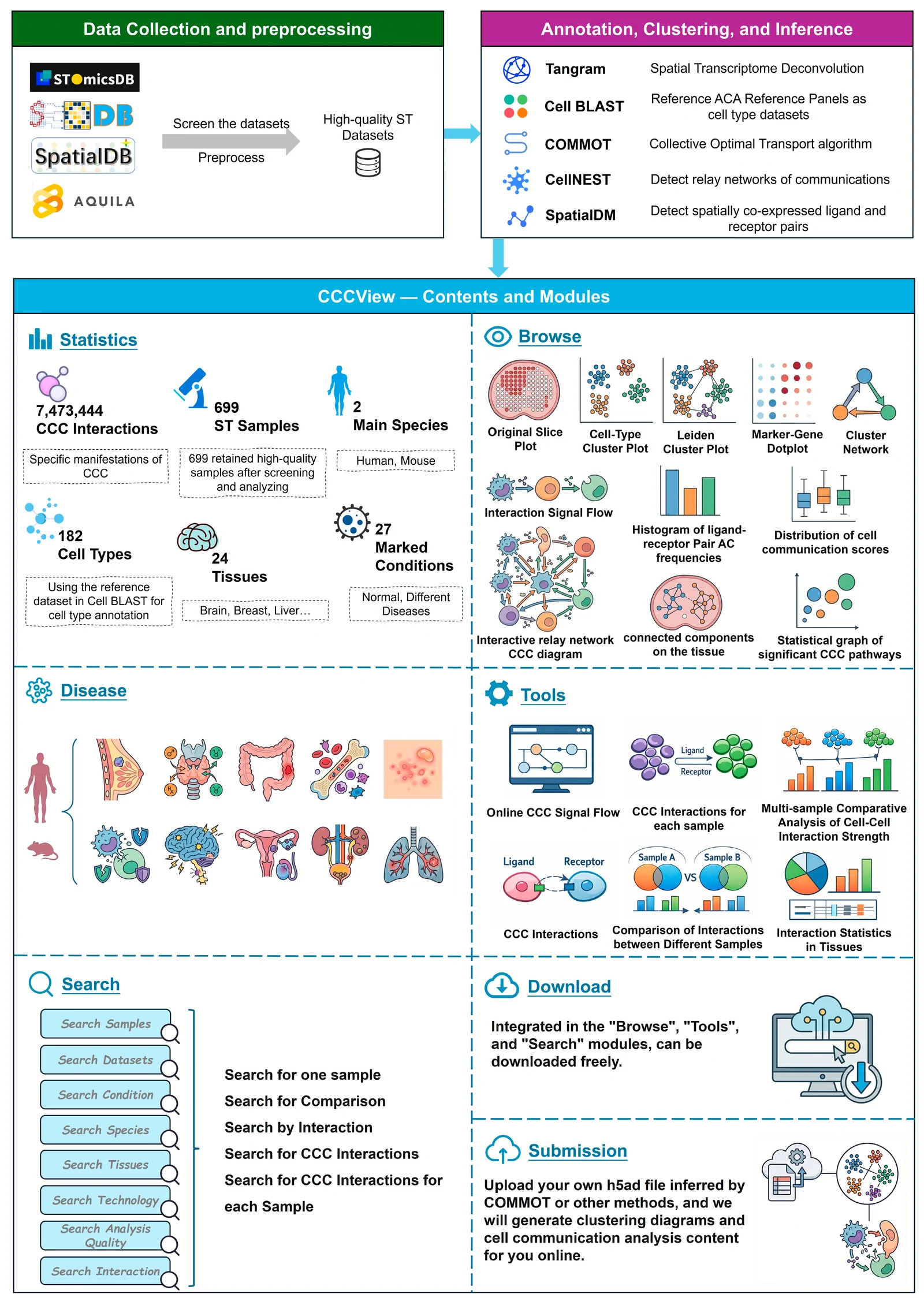
The entire process of CCCView from data collection, processing, analysis and inference, visualization, and development tools to web page display. The figure starts with data collection and preprocessing, outlining the data sources and preprocessing methods, followed by an overview of the clustering methods and inference algorithms used. Finally, the diagram outlines the various modules and their functions.

CCCView supports interactive querying of 7.4 million precomputed interactions through optimized indexing and back-end storage, enabling real-time exploration of large-scale CCC networks. Data include 24 tissue types, 182 cell types, and 27 marked conditions (normal and different diseases). Additionally, CCCView supports 12 visualization methods, five search strategies, six analysis and statistical tools, and four analytical algorithms to facilitate data analysis and exploration. The figure below shows the entire process of CCCView from data collection, processing, analysis and inference, visualization, and development tools to web page display (Figure 1).

### 2.1. Integrated Visualization of Spatial CCC Results

The Browse module provides sample-level access to spatial CCC results together with dataset metadata, including condition, species, tissue, technology, and analysis-quality information. Users can filter samples and inspect the corresponding spatial, cell-type, gene-expression, and communication views. The purpose of this module is to place inferred CCC patterns in the spatial and biological context of each source sample.

**Figure 2:**
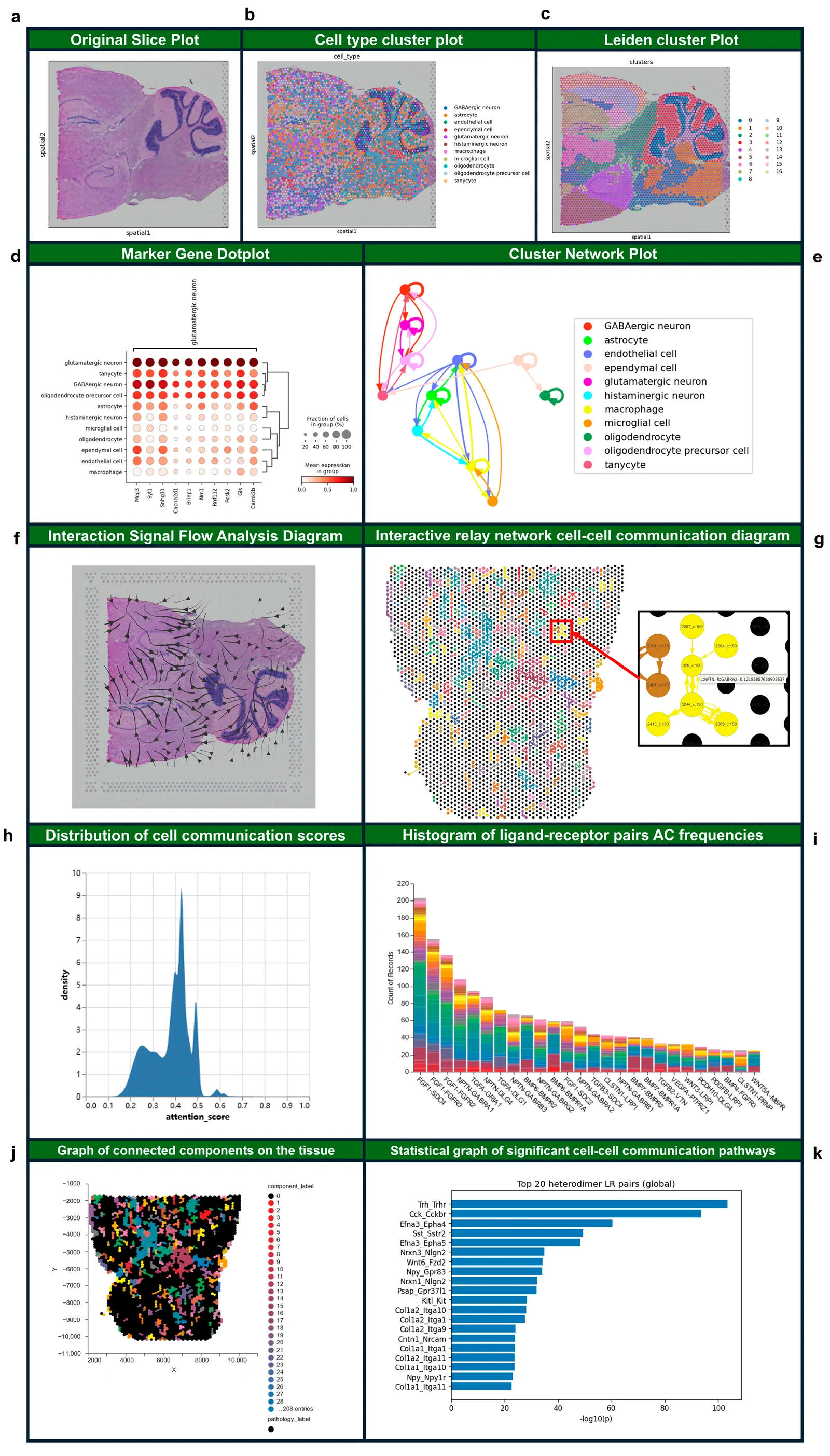
Visualization methods. (**a**) Example of original slice plot. (**b**) Example of cell-type cluster plot. (**c**) Example of Leiden cluster plot. (**d**) Example of marker-gene dotplot. (**e**) Example of cluster network plot. (**f**) Example of interaction signal flow analysis diagram. (**g**) Example of interactive relay-network cell–cell communication diagram. (**h**) Example of distribution of cell communication scores. (**i**) Example of histogram of ligand–receptor pair AC frequencies. (**j**) Example of graph of connected components on tissue. (**k**) Example of statistical graph of significant cell–cell communication pathways.

Twelve visualization types are available for complementary inspection of the processed data (Figure 2):

(1) Original Slice Plot: Displays the original morphology of the spatial transcriptomics section alongside CCC analysis results, allowing users to examine inferred communication patterns in their spatial context. This view provides the basis for most subsequent visualizations (Figure 2a).
(2) Dataset Information: Displays dataset metadata, sample information, and experimental methods for each sample.
(3) Cell-Type Cluster Plot: We display the clustering results after cell annotation using the spatial transcriptome deconvolution method and the CELL BLAST reference dataset. Each cell type is marked as an independent cluster, and different cell types are distinguished by color (Figure 2b).
(4) Leiden Cluster Plot: Uses the Leiden algorithm to identify clusters in spatial data, enabling comparison with the cell-type cluster plot (Figure 2c).
(5) Marker-Gene Dotplot: Based on the COMMOT analysis results, selects genes with significant expression differences in each cell-type cluster and plots the expression levels of these genes in each cluster. Through the size and depth of the points, we can intuitively show which genes play a leading role in a specific cell population. At the same time, we provide detailed data (Figure 2d).
(6) Cluster Network Plot: Based on the COMMOT analysis results, network graph analysis is performed based on cell-type clustering. The network graph shows the communication between cell populations and focuses on statistically significant pathways. The nodes in the graph represent cell populations that have passed through the cell-type clustering, and the edges represent the communication strength and direction, which helps users quickly understand the interaction pattern between cells. At the same time, we provide detailed data (Figure 2e).
(7) Interaction Signal Flow Analysis Diagram: This graph shows how cells send and receive information through specific signal pathways in the form of arrows. The size of the arrows is the signal strength and the direction is the signal transmission direction. This provides an intuitive way to observe and understand the communication between cells. We provide both the total results of all interactions and the results of online analysis of a single interaction. This helps users save on the huge computing power and time required for COMMOT analysis to infer spatial cell–cell communication, and they can quickly obtain the cell–cell communication signal flow diagram for each interaction under each sample (Figure 2f).
(8) Interactive Relay-Network Cell–Cell Communication Diagram: Zooming in on this diagram, presented in HTML format, reveals detailed chain information (including ligand–receptor pairs and strength), clearly showing the CCC network structure inferred by CellNEST (Figure 2g).
(9) Histogram of Ligand–Receptor Pair AC Frequencies: This histogram shows the frequency of communication via ligand–receptor pairs at the top 20% of attention edges with the highest scores (Figure 2i).
(10) Distribution of Cell Communication Scores: This graph shows the distribution density of attention scores. The horizontal axis represents attention_score, which indicates the importance or attention weight that the model assigns to each cell communication event. The vertical axis represents density, which reflects the frequency of different scores (Figure 2h).
(11) Graph of Connected Components on the Tissue: This image visualizes the cell communication connectivity components to which each spatial point in a tissue slice belongs, allowing us to observe the cell communication network structure and local communication module distribution inferred by CellNEST at the spatial level (Figure 2j).
(12) Statistical Graph of Significant Cell–Cell Communication Pathways: The most significant pathways in the sample, inferred from SpatialDM, were statistically analyzed (excluding L = R pathways) (Figure 2k).

### 2.2. Interactive Analysis and Cross-Sample Comparison

**Figure 3:**
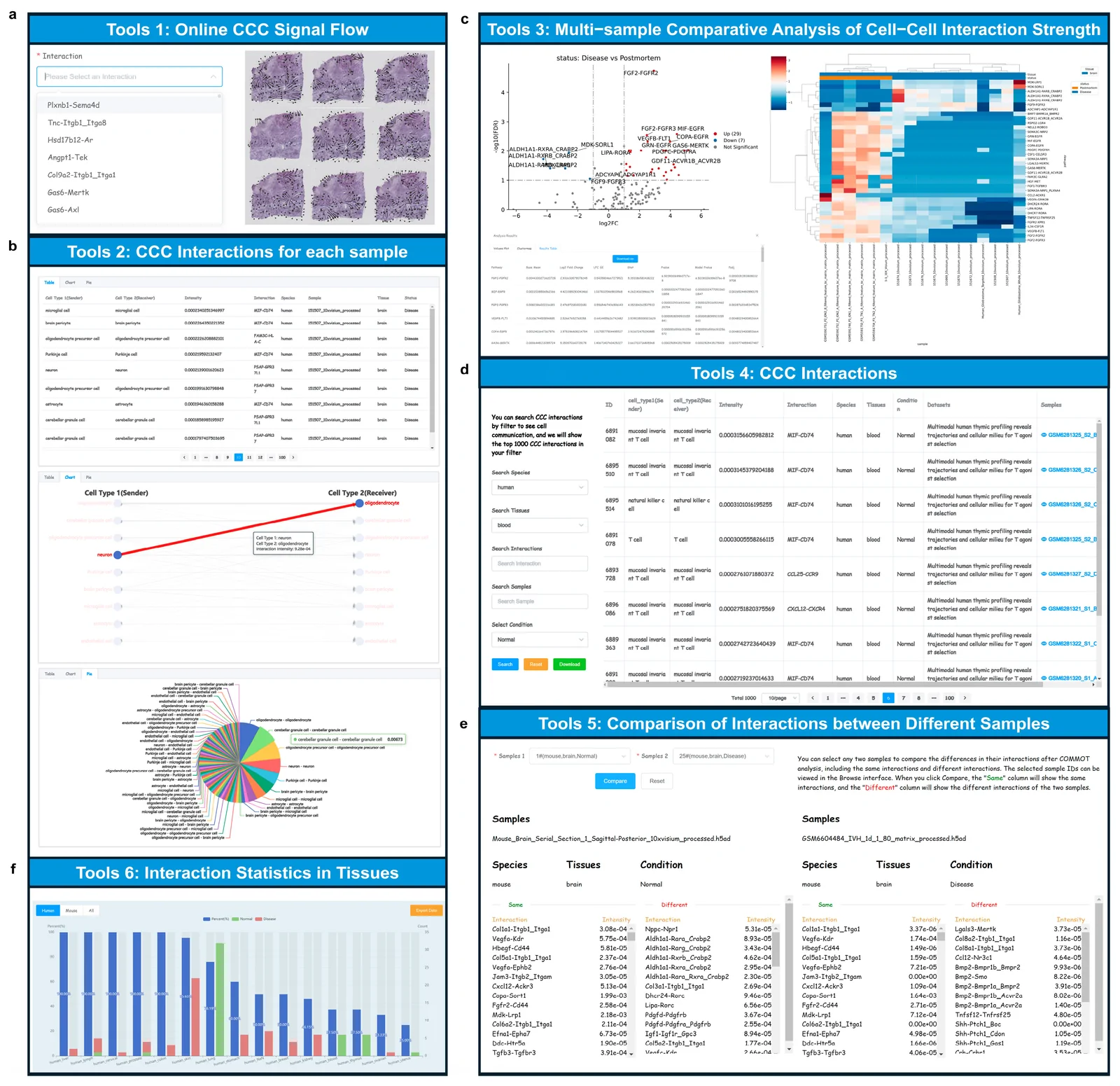
The “Tools” module. (**a**) Sample image example of Tool 1: Online CCC Signal Flow. (**b**) Interfaces of Tool 2: CCC Interactions for Each Sample, including the top 1000 interactions of the sample, the specific information on the total communication strength of each cell type as a sender and receiver, and the proportion of the total communication strength of each cell type as a sender and receiver to all communication strengths. (**c**) Example of Tool 3: Multi-sample Comparative Analysis of Cell–Cell Interaction Strength. (**d**) Main interface of Tool 4: CCC Interactions. (**e**) Analysis example of Tool 5: Comparison of Interactions Between Different Samples. (**f**) Analysis example of Tool 6: Interaction Statistics in Tissues.

The Tools module provides six functions for interrogating precomputed CCC results, ranging from single-sample inspection to cross-sample and multi-sample comparison (Figure 3). These tools expose the underlying interaction scores and summaries while reducing the need for users to rerun the full inference pipeline locally.

The six analysis and statistical tools are:

(1) Online CCC Signal Flow: To facilitate user analysis of cell–cell communication, we provide an online tool for quickly generating cell–cell communication signal flow diagrams for each CCC interaction of each sample. This tool greatly reduces the computing resource consumption of traditional COMMOT analysis, allowing users to quickly obtain detailed information on cell–cell communication (Figure 3a).
(2) CCC Interactions for Each Sample: For each sample, we have performed detailed statistical analysis of its CCC interactions. This tool can display the top 1000 interactions with the strongest cell–cell communication signal strength in the sample, the specific information on the total communication strength of each cell type as a sender and receiver, and the proportion of the total communication strength of each cell type as a sender and receiver to all communication strengths (Figure 3b).
(3) Multi-sample Comparative Analysis of Cell–Cell Interaction Strength: The quantification of cell–cell interactions in each sample can be decomposed into two core components: interaction strength and sender–receiver cell-type distribution patterns. This tool, exploiting the Tweedie regression model, performs differential strength analysis of multiple samples between two or more user-defined factor levels and provides multiple visualizations. After following the website prompts, the user can get the multi-sample comparative analysis results for cell–cell interaction strength, as shown in the figure below (Figure 3c).
(4) CCC Interactions: By selecting different conditions (species, tissues, condition, etc.), users can view the cell–cell communication interaction information under specific conditions, including the signal sender, receiver, intensity, etc., so that they can obtain more detailed cell–cell communication information and make in-depth comparisons. For example, we can select the three conditions “human”, “blood”, and “normal” to see the expression intensity of cell–cell communication interactions in this case (Figure 3d).
(5) Comparison of Interactions Between Different Samples: This tool enables users to compare the differences in cell–cell communication between two groups of samples. With this function, users can view the differences in cell–cell communication after COMMOT analysis, including changes in the interactions between different samples, as well as their intensity values, thereby helping to identify special communication patterns in disease states and identify potential biological features, especially the differences between disease samples and normal samples. For example, we selected sample 1, a normal mouse brain, and sample 25, a mouse brain with intraventricular hemorrhage. By comparing the samples with this tool, we can see the similarities and differences in cell–cell communication interactions and their signal strengths in different states, which will help us discover the impact of a disease on certain cell–cell communications (Figure 3e).
(6) Interaction Statistics in Tissues: We count the existing data, calculate the proportion of each interaction in different species and tissues, and show their differences. By generating a bar chart, users can quickly view the distribution of cell–cell communication in different species and tissues, further revealing the differences between normal and disease states (Figure 3f).

### 2.3. Search, Disease Annotation, Data Submission, and Documentation

The Search module supports retrieval by sample, dataset, condition, species, tissue, technology, analysis quality, and interaction. Search results link directly to the corresponding Browse and Tools views, allowing users to move from metadata filtering to sample-level inspection and comparative analysis.

The Disease module organizes curated disease-associated samples using anatomical-system categories informed by Disease Ontology [33]. The interface provides access to the corresponding samples and visualization results. These categories are intended for navigation and stratification of the resource and do not constitute independent disease re-annotation of the source studies.

The Submission module allows users to upload h5ad files containing results inferred by COMMOT or other compatible methods and obtain supported clustering and CCC visualizations. Sample data are provided for testing the workflow.

The Document module provides a user manual and descriptions of the algorithms, visualization functions, statistical analyses, and search functions implemented in CCCView. To further facilitate the use of the platform, we have provided a general step-by-step workflow (Supplementary Figure S8). Users can first identify samples or cell–cell communication interactions of interest through the Search or Browse modules using relevant metadata filters. They can then explore spatial cell-type distributions, ligand–receptor interactions, and inferred communication networks using the available visualization functions. For further investigation, the Tools module supports sample-level interaction statistics and cross-sample comparisons, including MuCIA-based analysis of interaction strength. Finally, users can interpret the resulting communication patterns in their biological context and download supported outputs for further analysis. This workflow is also included in the Document module to guide users through the main functions of CCCView.

### 2.4. Comparison with Existing Spatial Transcriptomics Resources

We compared CCCView with SpatialDB [34], STOmicsDB [22], CROST [35], Aquila [24], SPathDB [36], and SODB [23] using resource features related to CCC analysis (Table 1). CCCView differs from these broader spatial-omics resources by combining precomputed spatial CCC interactions, unified cell-type annotation, multiple CCC-inference methods, interactive CCC visualization, and multi-sample comparative analysis in a single platform. The comparison in Table 1 is feature-based and should not be interpreted as a benchmark of analytical accuracy or overall resource quality.

### 2.5. Case Study: Exploratory Analysis of Glioblastoma-Associated CCC

To illustrate how CCCView can be used for hypothesis-generating analysis, we examined a human glioblastoma spatial transcriptomics sample and then compared disease-associated and reference samples. This example is intended to demonstrate the platform workflow and biological interpretability of its outputs rather than to establish experimentally validated glioblastoma-specific communication mechanisms [37].

Using the Browse interface, human brain disease samples were retrieved and Human_Glioblastoma_Whole_10xvisium.h5ad (ID 196) was selected as an example. The cell-type view shows annotations generated by the Cell BLAST–Tangram workflow (Supplementary Figure S1), and the marker-gene dotplot provides an orthogonal transcriptional view of the annotated clusters (Supplementary Figure S2). In the cluster annotated as microglia, *C1QB* [38], *CD74* [39], *C3* [40], and *HLA-DRA* [41] were among the displayed genes. These genes are associated with complement, antigen-presentation, and immune functions that are compatible with a microglial transcriptional profile [42]. This concordance supports the plausibility of the annotation in this example, but it does not constitute an independent validation of cell identity.

The communication-network view for this sample highlights inferred interactions among multiple annotated cell populations (Supplementary Figure S3). Brain pericyte, microglial cell, and endothelial cell annotations appear prominently in the network. Because these labels are transferred through reference-based deconvolution, they should be interpreted as transcriptomic assignments rather than definitive anatomical identification. Detailed interaction scores and sender–receiver summaries can be inspected through the sample-level CCC tools and downloaded for further analysis.

We next used the platform’s multi-sample comparison function for an exploratory analysis of selected human brain samples, with disease status as the analysis condition and Postmortem as the reference level (Supplementary Figures S4 and S5). MDK-associated signals were increased in the analyzed disease group. The previous literature has implicated MDK in several malignancies, including glioblastoma [43,44,45]. This example illustrates how CCCView can combine cross-sample comparison with prior biological knowledge to prioritize communication patterns for follow-up. The observation should be regarded as hypothesis-generating and does not establish MDK as a diagnostic marker, therapeutic target, or causal driver in the analyzed samples.

As a further example, the MDK-SORL1 interaction was inspected with the Online CCC Signal Flow tool (Supplementary Figure S6). The resulting spatial view indicates regions in which the inferred interaction signal is concentrated and can be compared with cell-type annotations to generate hypotheses about potential sender and receiver populations. These patterns remain computational inferences and require independent experimental or orthogonal validation before biological conclusions are drawn.

### 2.6. Biological Consistency of Unified Cell-Type Annotations

To assess the biological consistency of the unified cell-type annotations, we examined two spatial transcriptomics datasets representing distinct tissue contexts, human heart and glioblastoma, using established cell-type marker genes (Supplementary Figure S7 and Table S1). As suitable author-provided spot-level cell-type annotations were unavailable for direct comparison in the selected datasets, we evaluated the expression patterns of canonical markers across predicted cell-type groups. Marker-expression consistency was assessed using dotplots and quantitative comparisons of marker detection rates and mean expression between each selected predicted cell-type group and the remaining spots. Unsupervised clustering assignments, such as Leiden clusters, were not considered ground-truth cell-type identities.

In the human heart dataset (4247 spots), several predicted cell populations exhibited marker-expression patterns consistent with their assigned identities. In particular, spots assigned to smooth muscle cells showed higher detection rates of *ACTA2* (53.40% versus 30.88%), *MYH11* (18.93% versus 6.73%), and *TAGLN* (48.06% versus 28.73%) compared with the remaining spots. Similarly, stromal cell assignments showed enrichment of *LUM* (72.06% versus 45.61%) and *COL1A1* (29.12% versus 11.54%). Vascular endothelial cell assignments also exhibited higher detection rates of *VWF* (76.38% versus 64.93%) and *PECAM1* (29.74% versus 24.51%), although the differences were less pronounced. By contrast, cardiac muscle-associated markers, including *TNNT2*, *MYH7*, and *ACTC1*, were broadly detected across multiple predicted groups, providing limited discriminatory power for evaluating individual cardiac muscle assignments. Endocardial markers also showed relatively weak differences between the corresponding predicted group and other spots. Overall, the heart dataset demonstrated clear marker-expression consistency for selected predicted populations, particularly smooth muscle and stromal cells, while the strength of marker support varied among cell types.

In the glioblastoma dataset (3468 spots), marker-expression analysis likewise revealed differences in transcriptional consistency across predicted cell-type groups. Several predicted neuronal and glial populations exhibited expression patterns broadly consistent with their associated markers, whereas some categories showed less distinct profiles. For example, spots assigned to the Purkinje cell category (n = 228) showed a higher detection rate of *GRID2* than the remaining spots (28.51% versus 5.59%). However, *PCP2* was not detected in this group, and *CALB1* and *ITPR1* showed limited discrimination. These observations indicate that the Purkinje cell assignments may reflect partial transcriptional similarity to the corresponding reference population rather than definitive identification of anatomically distinct Purkinje cells in glioblastoma tissue.

Taken together, these analyses demonstrate that the CCCView annotation workflow captures biologically relevant transcriptional features for several predicted cell populations, particularly smooth muscle and stromal cells in the heart dataset. Nevertheless, marker-expression consistency was not uniform across cell types, and some assignments lacked sufficient evidence for definitive cellular identification. Thus, the unified annotations provide biologically informative reference-based predictions, but their reliability remains dependent on cell type, tissue context, and the selected reference dataset.

## 3. Discussion

CCCView was developed to address a practical gap between the rapidly growing volume of spatial transcriptomics data and the fragmented landscape of CCC analysis resources. Its principal contribution is the integration of 699 samples into a harmonized, searchable knowledge platform containing 7,473,444 inferred interactions, together with unified cell-type annotation, complementary inference outputs, visualization, disease-oriented browsing, data access, and online analytical functions. By organizing these components within a common interface, CCCView enables users to move from a tissue or condition of interest to cell-type context, candidate ligand–receptor interactions, spatial communication patterns, and cross-sample comparisons without repeatedly reconstructing complete analysis workflows.

Several considerations are important when interpreting CCCView results. First, CCC interactions are computational inferences and do not by themselves demonstrate physical signaling or causal biological effects. Second, the source datasets were generated using different spatial transcriptomics technologies, resolutions, sequencing depths, and study designs. Although unified preprocessing reduces variability introduced by inconsistent analytical settings, it cannot eliminate platform- or study-specific differences. Because preprocessing results contribute to subsequent cell-type annotation and CCC inference, differences in gene detection sensitivity, data sparsity, and spatial resolution may propagate to downstream results, potentially affecting cell-type assignments and the number, distribution, and inferred strength of ligand–receptor interactions. Consequently, cross-sample differences may reflect both biological variation and technical effects. Comparisons between datasets with similar technical characteristics and biological contexts are generally more interpretable, whereas cross-platform comparisons should be regarded primarily as exploratory and evaluated in the context of the original study metadata. Third, Cell BLAST- and Tangram-based annotations are reference-dependent computational assignments and may reflect transcriptomic similarity rather than strict anatomical identity in some tissues. Fourth, COMMOT, CellNEST, and SpatialDM use different assumptions and score definitions. CCCView therefore preserves method identity rather than treating their outputs as directly interchangeable measurements. The platform’s cross-sample statistical module provides an additional exploratory comparison function, but its results are likewise dependent on the quality and comparability of the underlying inferred scores. Future development will focus on expanding data coverage, improving metadata harmonization and quality control, updating inference resources, and strengthening benchmarking of platform-level analytical functions and computational performance.

## 4. Materials and Methods

### 4.1. Data Collection and Preprocessing

Spatial transcriptomics datasets were collected from STOmicsDB [22], SODB [23], SpatialDB [34], and Aquila [24]. These resources were used as data sources rather than treated as mutually exclusive collections; consequently, datasets were screened before inclusion in CCCView. Candidate records from different source databases were cross-checked using original study identifiers, accession information, sample names, and source publications, and duplicate records corresponding to the same spatial transcriptomics sample were retained only once. We retained datasets containing spatial coordinates and gene-expression information that could be processed by the unified workflow described below. The objective of this curation step was to construct a harmonized collection suitable for cell-type annotation and CCC inference across heterogeneous ST studies.

Candidate datasets were evaluated for the availability and completeness of spatial coordinates, expression matrices, and metadata required for downstream processing. Datasets also had to be compatible with the preprocessing, dimensionality-reduction, cell-type annotation, and CCC-inference workflow. Samples passing these criteria were retained for analysis. Biological status information was manually curated and assigned to categories such as normal, disease, or other study-specific conditions to support subsequent stratified and comparative analyses. Because the source studies span different ST technologies and experimental designs, this harmonization improves comparability but does not eliminate platform- or study-specific effects.

For preprocessing, highly variable genes were selected using a coefficient-of-variation (CV) threshold greater than 0.5. Principal component analysis (PCA) was then performed and the first 50 principal components were retained. A k-nearest-neighbor graph was constructed, followed by UMAP with 15 neighbors and min_dist = 0.1 for low-dimensional visualization. A unified set of preprocessing parameters was applied across the 699 samples to establish a consistent and reproducible analytical framework for downstream cell-type annotation and CCC inference. This strategy was adopted to reduce additional variability arising from dataset-specific parameter choices, rather than to optimize preprocessing independently for each dataset. Although platform-specific optimization might better accommodate the characteristics of individual datasets, it would also introduce differences in analytical configurations that could complicate the interpretation of results across the integrated resource. Applying consistent parameters therefore provides a common methodological basis for processing heterogeneous datasets, while avoiding an additional source of variation introduced by independently selected preprocessing settings.

Nevertheless, methodological consistency does not imply technical equivalence among datasets. Differences in sequencing depth, gene detection sensitivity, and data sparsity may influence the genes retained under the same CV threshold and the information represented by the dimensionality-reduction results. Furthermore, differences in spatial resolution may affect the cellular composition represented by each spatial observation and the interpretation of spatial relationships. Because the preprocessing results are incorporated into subsequent Cell BLAST/Tangram-based cell-type annotation and CCC inference, these technical differences may propagate to downstream analyses, potentially affecting cell-type assignments, ligand–receptor detection, and the number, spatial distribution, and inferred strength of CCC interactions. Consequently, differences observed between samples may reflect both biological variation and technology- or study-specific effects. The unified preprocessing strategy was therefore adopted as a practical balance between large-scale methodological standardization and dataset-specific optimization, rather than as a means of eliminating technical heterogeneity. Cross-sample comparisons, particularly those involving different ST platforms, should be interpreted cautiously and in the context of the original technology, spatial resolution, sequencing depth, and biological background.

### 4.2. Cell-Type Annotation by Spatial Transcriptome Deconvolution Method

After preprocessing, we used the unified cell-type reference set Cell BLAST [32], which is a reference set traditionally used for single-cell RNA sequencing (scRNA-seq) data annotation. After that, we adopted the Tangram (v1.0.4) [31] spatial transcriptome deconvolution method, used the cell-type gene-expression features generated by Cell BLAST as the training gene set, integrated the single-cell transcriptome data and spatial transcriptome data through the Tangram tool, mapped these genes to the spatial transcriptome data, and compared the gene-expression patterns of the spatial locations with the expression profiles of the known cell types in the single-cell data, so as to infer the cell population composition of each spatial location and then realize the cell-type annotation in the spatial dimension.

For the training gene sets for each cell type, we selected the top 50 genes (n_top_genes = 50) from each cell type using the sc.get.rank_genes_groups_df() method to retrieve the top 50 highly expressed genes for each cell type. These genes were used to train the model for matching the spatial transcriptome data with the single-cell transcriptome data. To reduce data redundancy, we performed principal component analysis (PCA), retaining the top 50 principal components (n_pca_components = 50) for dimensionality reduction. Next, we used the UMAP method for further dimensionality reduction, setting 15 neighbors (n_neighbors = 15) and a minimum distance of 0.1 (min_dist = 0.1) to effectively visualize the high-dimensional data distribution. When annotating cell types in the spatial transcriptome data, we used the cell-type labels generated by Cell BLAST and mapped these labels to the spatial transcriptome data using the Tangram tool. We set a minimum expression threshold of 2% (perc = 0.02) for each cell type to ensure that annotated cell types have sufficient expression at spatial locations.

### 4.3. Cell–Cell Communication Inference

In the cell–cell communication inference process, we used three complementary algorithms: COMMOT [25], CellNEST [26], and SpatialDM [27].

To ensure consistency in ligand–receptor (LR) reference resources across the three CCC-inference methods, CellPhoneDB v4.0 was used as the common LR database for COMMOT, CellNEST, and SpatialDM. For human samples, LR pairs corresponding to human genes were used. For mouse samples, mouse-compatible LR pairs were obtained through COMMOT using the species parameter set to “mouse”, and the resulting LR list was subsequently provided as input to CellNEST and SpatialDM. Although the three methods used the same species-specific LR reference, their respective filtering criteria and inference procedures were retained. Consequently, the final sets of inferred interactions may differ between methods.

COMMOT is based on the Collective Optimal Transport (COT) model, which considers factors such as intercellular spatial distance and competition between ligands and receptors, quantifying the signal strength from the sending cell to the receiving cell. Ligand–receptor pairs were extracted from CellPhoneDB_v4.0 and filtered using ct.pp.filter_lr_database(min_cell_pct = 0.05), retaining only pairs expressed in ≥5% of cells. Communication inference used ct.tl.spatial_communication() with a distance-threshold parameter of dis_thr = 500, restricting the analysis to spatial observations within 500 coordinate units; setting heteromeric = True enabled the consideration of multimeric ligand–receptor complexes, and enabling pathway_sum = True summarized signal strengths from multiple pathways.

The distance-threshold parameter was fixed at dis_thr = 500, consistent with an example setting provided in the official COMMOT tutorial, to maintain a common computational configuration across the integrated datasets. This choice was motivated by the need to support systematic cross-sample exploration while avoiding additional variability introduced by dataset-specific parameter selection. However, the applicability of a fixed numerical threshold depends on the characteristics of the spatial data. Different ST technologies may use different coordinate scales, and variations in spatial resolution may affect the density and distribution of spatial observations. In addition, differences in tissue architecture and cellular organization may influence the biologically relevant range of communication. Because the original spatial coordinates were used without conversion to a common physical unit, dis_thr = 500 does not necessarily correspond to the same physical distance across datasets. Accordingly, this parameter represents a standardized computational setting rather than a universally optimal biological signaling distance, and its potential influence on cross-sample comparisons should be considered when interpreting COMMOT-derived results.

CellNEST is a deep learning-based method for resolving complex relay-network communication patterns in spatial transcriptome data. Unlike traditional methods that can only detect single ligand–receptor pairs and often have high false positive rates, CellNEST can identify ligand–receptor–ligand–receptor chain signals, revealing more advanced intercellular communication patterns. Spatial transcriptome data, after preprocessing, is used to train the model in a Singularity container with five independent runs (run_id = 1–5), each training for 500 epochs using a fixed random seed to ensure reproducibility. Post-processing integrates all run results to generate robust cell-type predictions and communication signal matrices, and visualizes the top 3000 strongest interactions.

SpatialDM is a spatially aware statistical method for ligand–receptor interaction inference. It calculates the global and local spatial significance of LR pairs and estimates intercellular signal intensity. The analysis process included: calculating the effective distance (k = 8) using nearest neighbors and constructing a weight matrix (cutoff = 0.1); extracting species-specific LR pairs from the shared CellPhoneDB v4.0 reference (minimum expression threshold of 3 cells); evaluating global and local significance (z-score, FDR ≤ 0.1); and screening heterologous ligand–receptor pairs, etc. The spatial distribution and meta-information of individual LR pairs were recorded simultaneously to ensure reproducibility of the analysis.

The outputs from COMMOT, CellNEST, and SpatialDM are stored in a unified interaction schema. Cross-method consistency is assessed through side-by-side comparison of ligand–receptor-level interaction patterns rather than numerical aggregation. These methods are not merged into a single score but provide complementary perspectives for ligand–receptor-based, spatial-statistical, and relay-network communication inference.

### 4.4. Visualization Methods and Statistical Analysis Tools

After inferring cell–cell communication and annotating cell types, we developed a series of data visualization methods and statistical analysis tools to clearly and intuitively display the communication between cells and assist in further analysis. The visualization methods we developed and applied include the original slice plot, clustering diagrams for cell clustering using Leiden and deconvolution methods, dotplots that reflect the expression levels of genes in each cluster, network diagrams that show the communication between cell populations and their interaction patterns, signal flow diagrams that intuitively show the communication between cells, interactive relay-network cell–cell communication diagrams, etc. The analysis and statistical tools we developed can perform online analysis on the communication signal flow graph of each cell–cell communication interaction, perform conditional, comparative and statistical analysis on the sender, receiver and intensity of cell–cell communication interactions under different conditions, select different categories of samples for differential analysis of cell–cell communication, query the proportion of different cell–cell communication interactions under different conditions, etc.

### 4.5. Multi-Sample Cell–Cell Communication Integrated Analysis Method (MuCIA)

#### 4.5.1. MuCIA Model Specification

The cell-type-aggregated connectome of *n* samples with *q* cell types (cell clusters) and *m* cell–cell interactions is formulated as a fourth-order tensor, $C_{\mathrm{nmkk}}\in R^{n\times m\times q\times q}$, with each element *c_ijkl_* denoting the non-negative interaction score of interaction *j* between sender cell type *k* and receiver cell type *l* in sample *i*. Underlying this framework, observed scores *c_ijkl_* are assumed to arise from a compound Poisson–gamma (Tweedie) distribution. Specifically, each score represents the sum of $n_{\mathrm{ijkl}}^{+}$ independent gamma-distributed cell-pair interaction scores, where $n_{\mathrm{ijkl}}^{+}$ follows a Poisson distribution.

MuCIA uses ligand–receptor communication scores inferred by COMMOT as its upstream input. Each score is indexed by sample, ligand–receptor pair, sender cell type, and receiver cell type. For a given sample and ligand–receptor pair, the scores are summed over sender–receiver cell-type combinations to obtain the sample-level interaction strength; the pair-specific scores are retained separately to estimate sender–receiver interaction distributions.

Each Tweedie distribution is characterized by three parameters: power index *p ϵ* (1,2), mean *μ* > 0, and dispersion *σ*^2^ > 0. Estimation of parameters for all interaction and sender–receiver pair combinations separately introduces substantial statistical challenges due to:

1. High parameter dimensionality (O(mq^2^)) exceeding typical sample size (n ≪ mq^2^).

2. Loss of statistical power from multiple hypothesis correction.

There is currently no universal ground-truth benchmark for spatial cell–cell communication, which motivates the use of a comparative rather than predictive modeling framework.

The characterization of each interaction involves two key aspects: its interaction strength and the distribution of signals across sender–receiver cell-type pairs. Accordingly, we separate interaction strength from sender–receiver distribution:

Strength matrix $S\in R^{n\times m}$ aggregates interaction scores:

(1) $$s_{\mathrm{ij}}=\sum_{k}\sum_{l}c_{\mathrm{ijkl}},$$

(2) $$s_{\mathrm{ij}}\sim \mathrm{Tw}\left(\mu_{\mathrm{ij}},\;\sigma_{j}^{2};p_{j}\right).$$

Distribution weight tensor $W\in R^{m\times q\times q}$ captures sender–receiver allocation:

(3) $$c_{\mathrm{ijkl}}\sim w_{j\mathrm{kl}}\mathrm{Tw}\left(\mu_{\mathrm{ij}},\frac{\sigma_{j}^{2}}{w_{j\mathrm{kl}}};p_{j}\right)\;s.t.0\leq w_{\mathrm{jkl}}\leq 1,\;\sum_{k}\sum_{l}w_{\mathrm{jkl}}=1.$$

For each interaction j, a Tweedie generalized linear model relates interaction strength to variables of interest:

(4) $$\log_{2}\left(\mu_{j}\right)=X\beta_{j}+\epsilon_{j},$$

where ***X*** denotes the design matrix. ***β****_j_* and ***ϵ****_j_* denote the coefficient vector and random error vector of interaction *j*, respectively. Estimation and Wald testing on each ***β****_j_* filter out interactions with variable strength between levels of the variable of interest in ***X***. Power parameters *p_j_* are estimated via ordinary least squares (OLS) regression of the log-variance versus log-mean relationship.

Implementation clarification: The Tweedie variance-power parameter is estimated as the slope of an ordinary least squares (OLS) regression of log variance on log mean across interactions, rather than by ordinary least squares (OLS) regression. A common estimated power parameter is supplied to the interaction-specific Tweedie generalized linear models (GLMs). The fitted GLM uses a natural-log link, and the estimated two-group coefficient is divided by ln(2) to obtain the log2 fold change. Locally weighted scatterplot smoothing (LOWESS) is used for selecting the independent-filtering threshold, not for estimating the Tweedie power parameter.

(5) $$\ln\left(v_{j}\right)=p_{j}\ln\left(\mu_{j}\right)+\ln(\sigma^{2}).$$

For zero-value handling, sample–interaction combinations absent from the aggregated strength matrix are filled with zeros. Interactions with positive strengths in fewer than two samples are excluded from differential testing, whereas zero observations for retained interactions are preserved. For multiple testing, MuCIA adjusts Wald-test *p*-values using the Benjamini–Hochberg (BH) procedure, together with mean-based independent filtering. Candidate filtering thresholds are evaluated according to the number of discoveries, with LOWESS smoothing used to select a threshold; filtered interactions receive no adjusted *p*-value. The analysis uses an adjusted *p*-value cutoff of 0.10.

Maximum likelihood estimation of each ***W****_j_*, the sender–receiver distribution matrix of interaction *j*, is performed on ${\hat{\mu }}_{j}$ and ${\hat{\sigma }}_{j}^{2}$ estimated by the above Tweedie regression model. Extreme value distribution is exploited to test whether an interaction was enriched in some sender–receiver pair or distributed equally. Hellinger distance is used to explore the distribution similarity of interactions. MuCIA is designed as a statistical comparative framework rather than a supervised predictive model; therefore, it does not aim to establish ground-truth validation of CCC events.

#### 4.5.2. CCI Interaction Score Normalization

The raw scores supplied to the normalization procedure are the sender–receiver-specific ligand–receptor communication scores inferred by COMMOT.

The raw interaction scores are normalized to minimize the total variance in interaction strengths before building the MuCIA model, ensuring scale-invariant comparisons across samples:

(6) $$c_{\mathrm{ijkl}}=\frac{c_{\mathrm{ijkl}}^{\prime }}{a_{i}},$$

(7) $$a=\mathrm{argmi}n_{a}(\mathrm{tr}({\left(S-{\frac{1}{n}1}_{\mathrm{nn}}S\right)}^{T}\left(S-{\frac{1}{n}1}_{\mathrm{nn}}S\right)),\;s.t.a_{i}>0,\;\sum^{i}{\ln(a}_{i})=0,$$

where *a_i_* is the size factor of sample *i*, $c_{\mathrm{ijkl}}^{\prime }$ is the raw interaction score and *c_ijkl_* is the normalized score.

The same sample-specific size factor is applied to all COMMOT-derived ligand–receptor scores and sender–receiver cell-type pairs. Consequently, relative sender–receiver contributions within each sample and interaction are preserved after normalization. The normalized interaction strengths are used for differential analysis, while normalized sender–receiver-specific scores are retained for interaction-distribution estimation.

#### 4.5.3. Simulation-Based Evaluation of MuCIA

To evaluate MuCIA under known group effects, synthetic non-negative ligand–receptor interaction strengths were generated from a compound Poisson–gamma process corresponding to a Tweedie distribution, which permits exact zero observations. Each dataset comprised 100 ligand–receptor interactions across two groups, with 20% assigned a predefined effect and the remaining interactions generated under the null hypothesis. We examined sample sizes of 10, 20, and 30 per group; true log2 fold changes of 0.5, 1.0, and 1.5; and dispersion parameters of 0.5, 1.0, and 2.0. A global-null scenario without true group effects was also included. Each condition was repeated 100 times.

The generated strengths were processed using the MuCIA statistical workflow, including minimum-variance normalization, Tweedie generalized linear modeling, Wald testing, mean-based independent filtering, and BH-adjusted *p*-values at a threshold of 0.10. The empirical FDR was defined as the average false discovery proportion across simulation replicates; power was the fraction of truly differential interactions detected; and effect-size bias was calculated from estimated minus true log2 fold changes. Model-fitting failures were recorded separately.

The simulation outcomes are summarized in Supplementary Table S2. Across the sample-size conditions, the empirical FDR ranged from 0.071 to 0.082, and power increased from 0.480 at n = 10 to 0.860 at n = 30. Under true log2 fold changes of 0.5 and 1.5, the corresponding powers were 0.390 and 0.910, respectively. The empirical FDR under the global-null condition was 0.040.

### 4.6. Database Implementation

CCCView was developed using a front-end and back-end architecture. The front end was implemented using Vue 3 (https://vuejs.org/), with HTML and CSS for page structure and styling. Element UI (https://element.eleme.io/, accessed on 2 August 2025) was incorporated to provide reusable interface components and support interactive user operations. The back end was developed using Django (v5.2.4) (https://www.djangoproject.com/, accessed on 2 August 2025), which handles server-side logic, data processing, and database operations. Django’s built-in Object-Relational Mapping (ORM) framework facilitates data management and querying, while the Django REST Framework (v3.16.0) (https://www.django-rest-framework.org/, accessed on 2 August 2025) enables communication and data exchange between the front end and back end.

SQLite (https://www.sqlite.org/, accessed on 2 August 2025) is used as the database back end in the deployed CCCView application. To accommodate different types of data, CCCView adopts a hybrid storage architecture that combines SQLite with file-based storage. Textual and structured information is stored directly in SQLite, whereas image files and other large data resources are maintained separately in the server’s file system, with their corresponding file paths or references recorded in the database. This architecture separates structured data management from the storage of large file-based resources.

CCCView is deployed on a server running Debian GNU/Linux 12, equipped with eight virtual CPUs (Intel Xeon Gold 6330, 2.00 GHz), approximately 125 GiB of RAM, and a 2 TB virtual disk. The platform is accessible through a university-managed network, for which no fixed guaranteed public-facing bandwidth is specified.

To evaluate the loading performance of interactive visualizations, we conducted a practical assessment using the Browse and Tools modules. Loading time was defined as the interval from clicking a sample or visualization button to the point at which the complete visualization was displayed and ready for interaction. Under single-user access, the tested visualizations completed loading within approximately 5 s. In a simultaneous-access test involving five users, each using a separate computer to open the same visualization page, loading was also completed in approximately 5 s. Once the required resources had been loaded, subsequent interactions within the same page did not require reloading those resources. These observations reflect performance under the tested conditions rather than a comprehensive large-scale concurrency benchmark.

### 4.7. User Data Upload, Analysis, and Data Management

The CCCView user data upload module accepts AnnData (.h5ad) files with a maximum size of 500 MB per submission. Each submission is restricted to one sample, and the corresponding input requirements are provided on the upload interface. Under typical usage conditions, the analysis is completed within approximately 1–2 min, depending on dataset size, complexity, and server workload.

Uploaded datasets are processed using the computational resources of the CCCView server, which is equipped with eight virtual CPUs and approximately 125 GiB of RAM. The file-size and single-sample restrictions define the supported input limits. Multiple analysis tasks can execute concurrently when users submit datasets simultaneously, with each user independently awaiting their analysis results. Processing times may increase under higher computational loads or greater numbers of simultaneous submissions.

Uploaded files are stored on the server and automatically deleted after a retention period of 24 h. Uploaded data are not accessible to other users, thereby limiting data retention and restricting cross-user access.

## Figures and Tables

**Table 1:** Comparison between CCCView and other spatial transcriptome databases and knowledge platforms.

	CCCView	StomicsDB	CROST	Aquila	SPathDB	SODB
CCC interactions	7,473,444	-	-	-	114,998	-
Annotated cell types	182	-	168	-	-	-
Ligand–receptor analysis	√	√	-	√	√	-
Cell clustering	√	√	-	-	√	-
Cell-type annotation	√	-	-	-	-	-
Cell–cell cluster communication analysis	√	-	√	-	-	-
Spatial CCC identification	√	√	-	-	√	-
Spatial CCC inference	√	-	-	-	-	-
Spatial CCC visualization	√	√	-	-	-	-
Online CCC analysis	√	-	-	√	√	-
Multi-sample comparative analysis of cell–cell interaction strength	√	-	-	-	-	-

√, feature reported in the cited publication/resource; -, not reported in the cited publication/resource at the time of comparison.

## Data Availability

CCCView is available at http://cccview.bjmu.edu.cn/. The platform provides access to the processed cell–cell communication results and associated sample information described in this study.

## Supplementary Materials

The following supporting information can be downloaded at https://www.mdpi.com/article/10.3390/ijms27209382/s1.
