# Supplementary material for: CCCView: A Knowledge Platform for Integrated Analysis of Spatial Transcriptomic Cell–Cell Communication
Source: Int J Mol Sci. 2026 Oct 21;27(20):9382. doi: 10.3390/ijms27209382 (PMC13704988; doi:10.3390/ijms27209382)
Supplement: Supplementary file 1 [file ijms-27-09382-s001.zip › ijms-4610583-supplementary/Supplementary Figure S1.pdf]

Supplementary Figure S1. Cell type cluster plot of "Human_Glioblastoma_Whole_10xvisium"

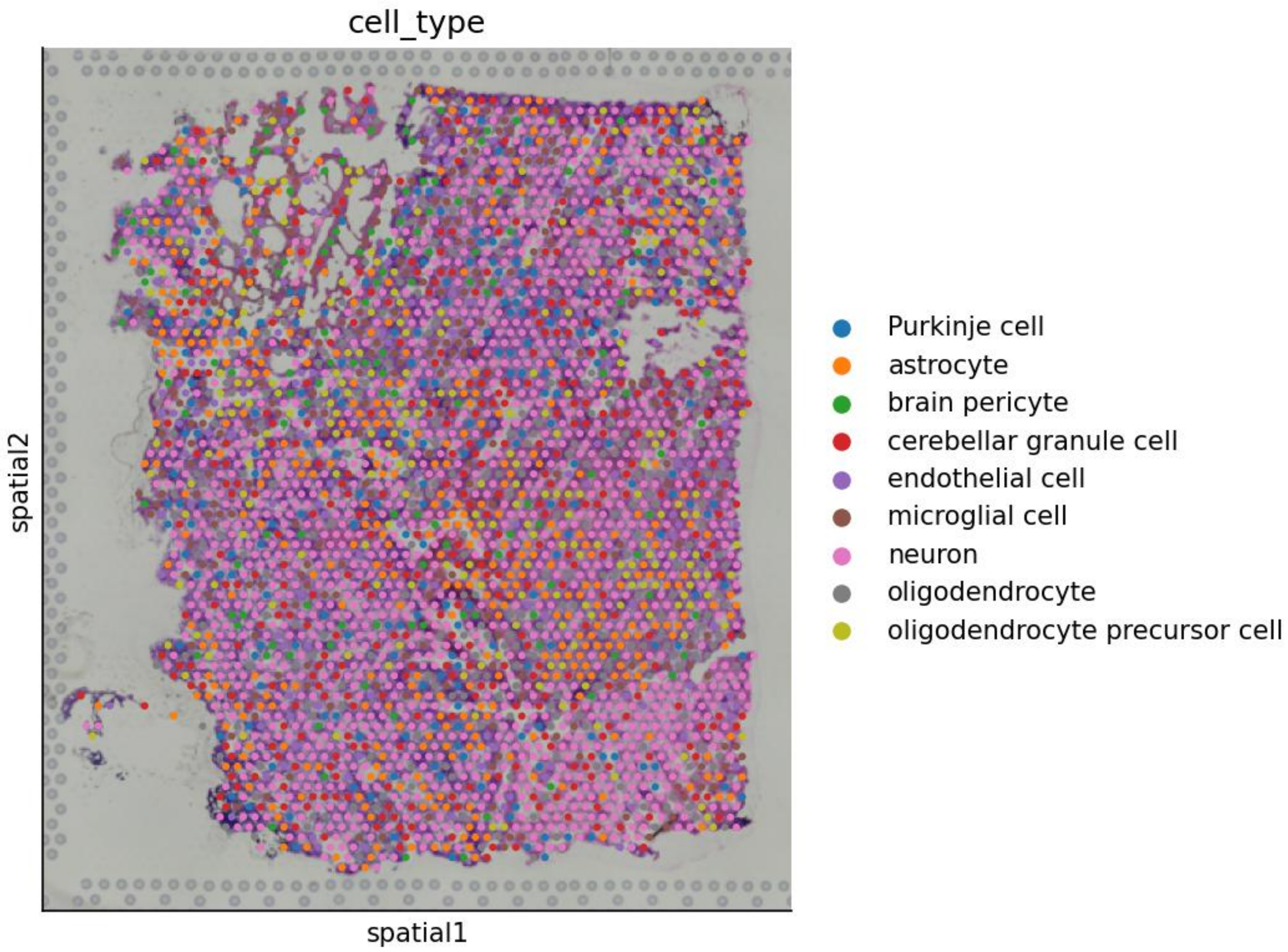

Supplementary Figure S2. Marker Gene Dotplot of “Human_Glioblastoma_Whole_10xvisium”

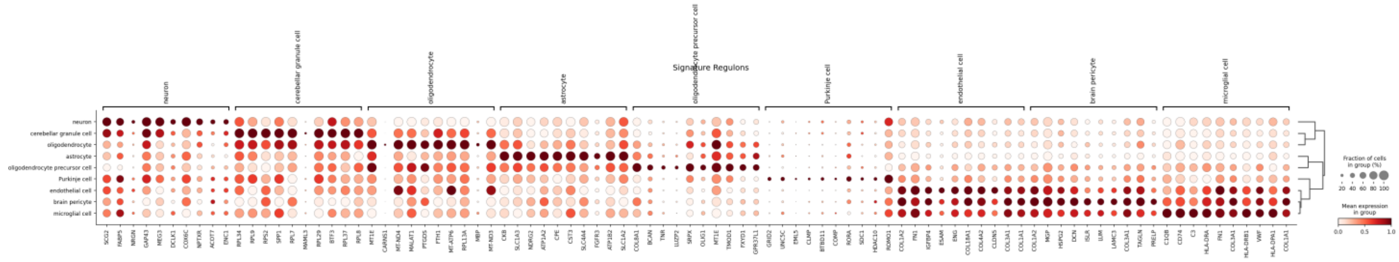

Supplementary Figure S3. Cell cluster-cluster communication Network diagram of "Human_Glioblastoma_Whole_10xvisium"

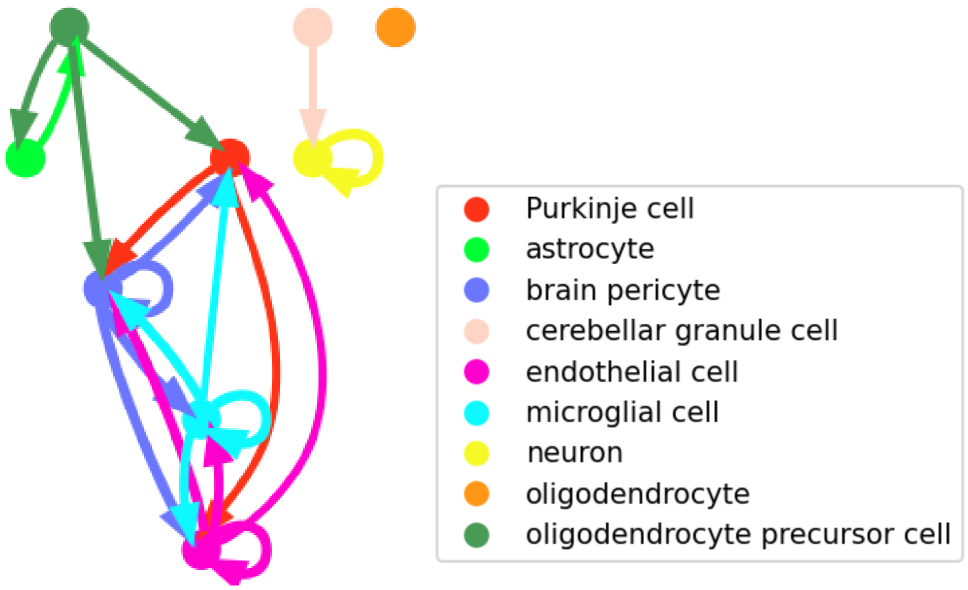

Supplementary Figure S4. Volcano map of analysis results

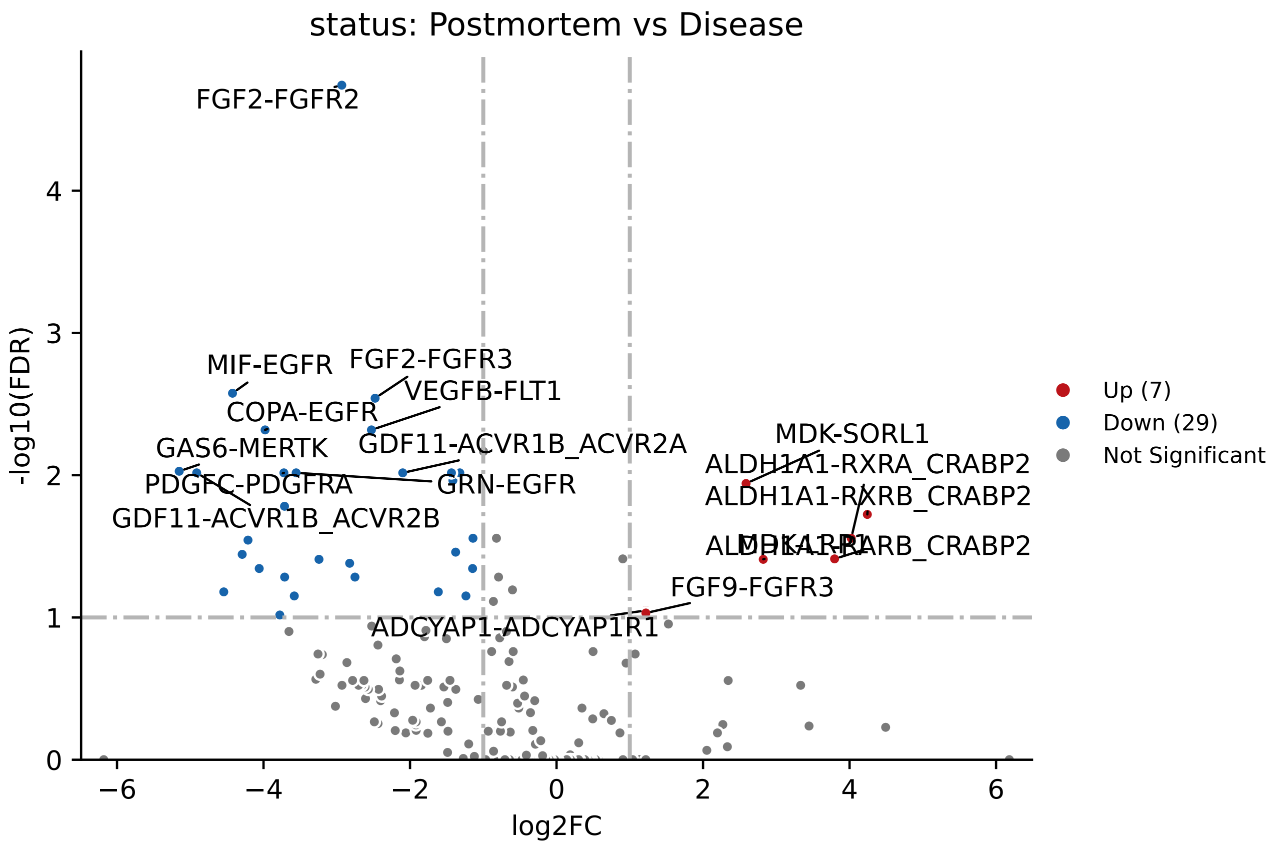

Supplementary Figure S5. Cluster map of analysis results

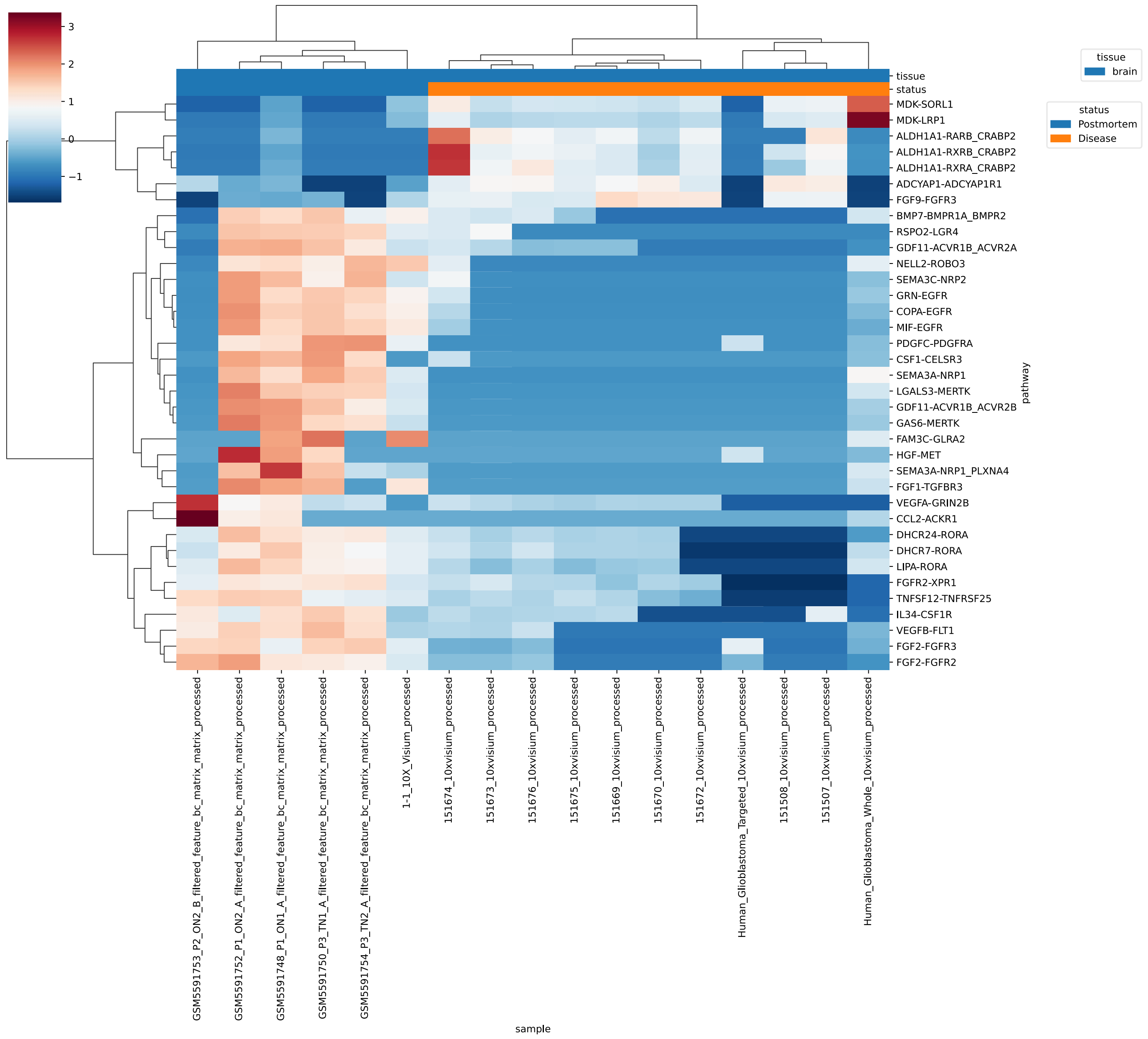

Supplementary Figure S6. CCC Singal Flow diagram of “Human_Glioblastoma_Whole_10xvisium”

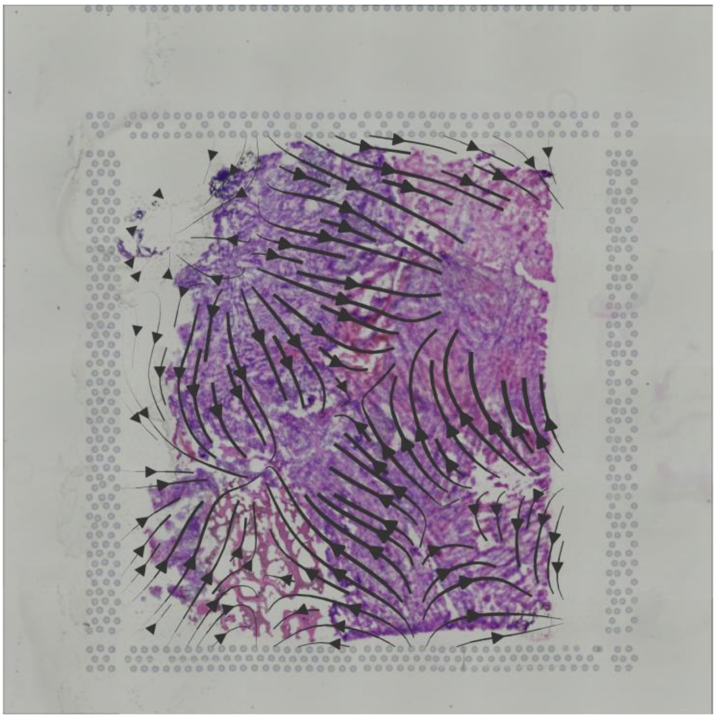

Supplementary Figure S7. **Marker-expression consistency of unified cell-type annotations in representative spatial transcriptomics datasets.** (A) Dot plot showing the expression of selected cardiac muscle, vascular endothelial, smooth muscle, stromal, and endocardial markers across predicted cell-type groups in the human heart dataset (4,247 spots). (B) Dot plot showing the expression of selected neuronal, astrocytic, oligodendrocytic, microglial, endothelial, and Purkinje-associated markers across predicted cell-type groups in the glioblastoma dataset (3,468 spots). Dot size indicates the percentage of spots with detectable expression of each gene, and dot color indicates mean expression scaled independently for each gene across predicted cell-type groups. The cell-type labels represent reference-based transcriptomic assignments. The “nan” category in the heart dataset corresponds to an unannotated category inherited from the single-cell reference.

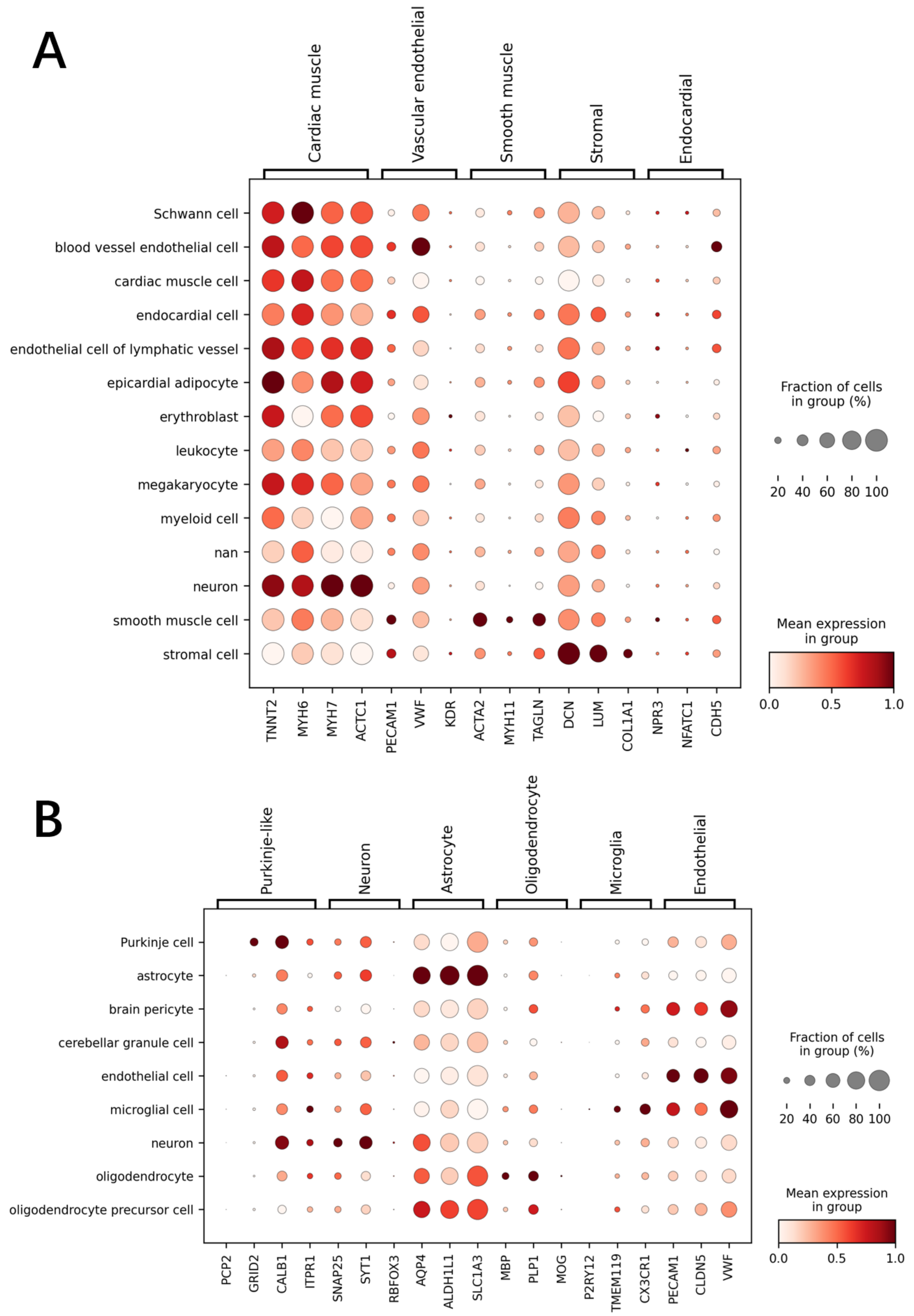

Supplementary Figure S8. General workflow of the CCCView platform.

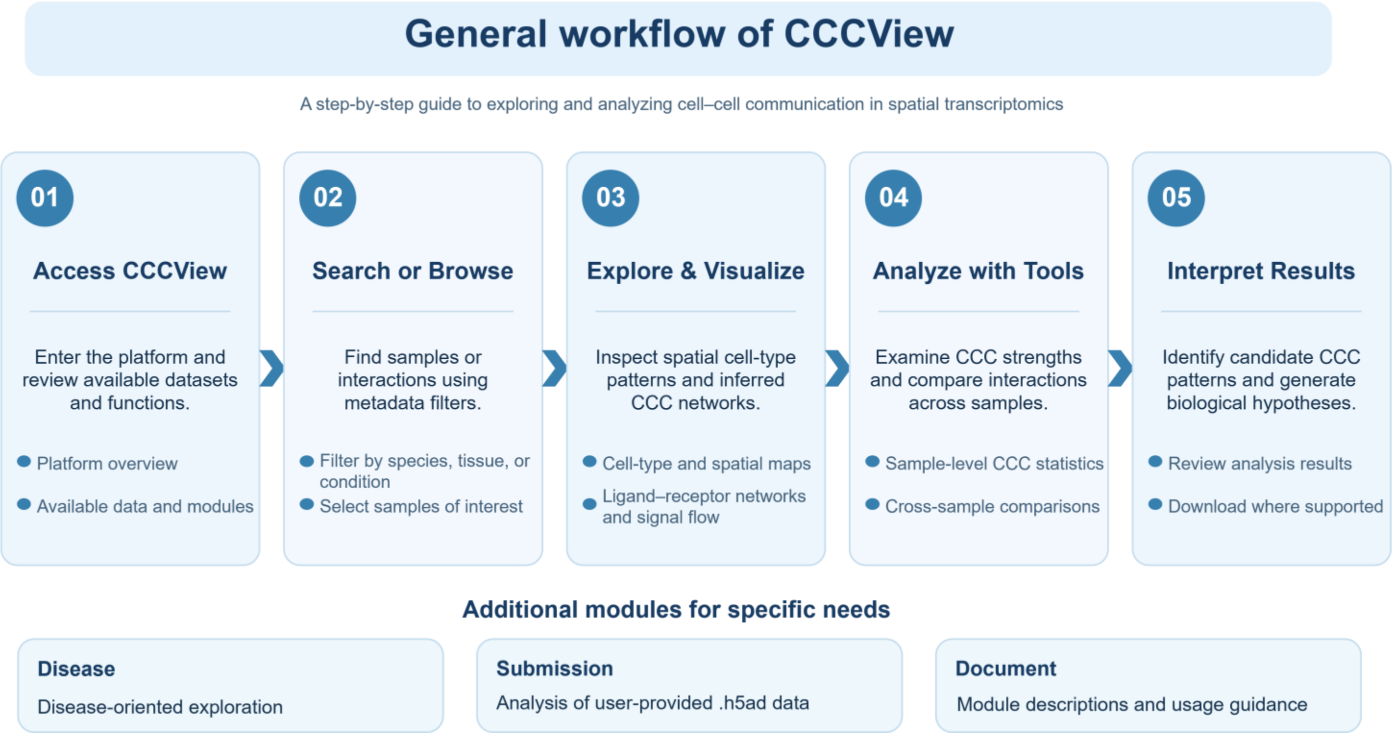

Supplementary Table S1. Quantitative assessment of marker-expression consistency across selected predicted cell-type groups in human heart and glioblastoma spatial transcriptomics datasets.

For each cell-type–marker pair, the table reports the number of spots assigned to the corresponding predicted group, the percentage of spots with detectable marker expression, and the mean expression level in the predicted group and all remaining spots.

Supplementary Table S2. Simulation-based assessment of MuCIA

Supplementary Table S3. Comprehensive sample metadata and data provenance of spatial transcriptomics datasets included in CCCView.
