# Supplementary material for: CCCView: A Knowledge Platform for Integrated Analysis of Spatial Transcriptomic Cell–Cell Communication
Source: Int J Mol Sci. 2026 Oct 21;27(20):9382. doi: 10.3390/ijms27209382 (PMC13704988; doi:10.3390/ijms27209382)
Supplement: Supplementary file 1 [file ijms-27-09382-s001.zip › ijms-4610583-supplementary/Supplementary_Table_S2.pdf]

Supplementary Table S2. Simulation-based assessment of MuCIA

Observed results from 100 independent simulation replicates per scenario (nominal BH-adjusted P-value threshold = 0.10).

Simulation condition	Empirical FDR	Power	Log2FC bias	Zero fraction	Successful runs
Sample size: 10/group	0.082	0.480	-0.060	0.085	100/100
Sample size: 20/group	0.076	0.720	-0.040	0.085	100/100
Sample size: 30/group	0.071	0.860	-0.020	0.085	100/100
Effect: log2FC = 0.5	0.069	0.390	-0.030	0.088	100/100
Effect: log2FC = 1.0	0.076	0.720	-0.040	0.085	100/100
Effect: log2FC = 1.5	0.084	0.910	-0.050	0.082	100/100
Dispersion: 0.5	0.064	0.830	-0.020	0.012	100/100
Dispersion: 1.0	0.076	0.720	-0.040	0.085	100/100
Dispersion: 2.0	0.089	0.540	-0.070	0.275	100/100
Global null (no true effects)	0.040	N/A	N/A	0.090	100/100

Empirical FDR denotes the mean false discovery proportion across replicates; power is the proportion of truly differential interactions detected; log2FC bias is estimated minus true log2 fold change. Zero fraction denotes the proportion of zero-valued strengths.
